# Augmenting the Transplant Team With Artificial Intelligence: Toward Meaningful AI Use in Solid Organ Transplant

**DOI:** 10.3389/fimmu.2021.694222

**Published:** 2021-06-11

**Authors:** Jeffrey Clement, Angela Q. Maldonado

**Affiliations:** ^1^ Information and Decision Sciences, Carlson School of Management, University of Minnesota, Minneapolis, MN, United States; ^2^ Scientific Affairs, Hansa Biopharma AB, Lund, Sweden

**Keywords:** artificial intelligence, machine learning, natural language processing, decision making, shared decision model, transplant, immunosuppression, ethics

## Abstract

Advances in systems immunology, such as new biomarkers, offer the potential for highly personalized immunosuppression regimens that could improve patient outcomes. In the future, integrating all of this information with other patient history data will likely have to rely on artificial intelligence (AI). AI agents can help augment transplant decision making by discovering patterns and making predictions for specific patients that are not covered in the literature or in ways that are impossible for humans to anticipate by integrating vast amounts of data (e.g. trending across numerous biomarkers). Similar to other clinical decision support systems, AI may help overcome human biases or judgment errors. However, AI is not widely utilized in transplant to date. In this rapid review, we survey the methods employed in recent research in transplant-related AI applications and identify concerns related to implementing these tools. We identify three key challenges (bias/accuracy, clinical decision process/AI explainability, AI acceptability criteria) holding back AI in transplant. We also identify steps that can be taken in the near term to help advance meaningful use of AI in transplant (forming a Transplant AI Team at each center, establishing clinical and ethical acceptability criteria, and incorporating AI into the Shared Decision Making Model).

## Introduction

### Background and Motivation

Research into artificial intelligence (AI) applications in transplant have identified ways that it could influence better outcomes for patients by potentially facilitating an increase in the number of transplants, improving matching between donors and recipients, personalizing medication regimens, or assessing risk of patient nonadherence, to name just a few possibilities (1, 2). AI agents can help augment transplant decision making in two primary ways. First, AI can discover patterns and make predictions for specific patients that are not addressed in the literature, or covered by protocols (e.g., accounting for comorbidities) or in ways that are impossible for humans to anticipate (e.g., modeling pharmacokinetics). Second, similar to other clinical decision support systems, AI can help overcome human biases or judgment errors (3). However, AI does not always outperform traditional statistical techniques (e.g., linear regression, Kaplan Meier survival analysis) (4–6). AI is not widely utilized in transplant to date— AI tools such as those reported in the papers in our review are used in a research settings in transplant, but are not widely used. In contrast with some other fields (e.g. radiology), no transplant AI tools have progressed to the point of FDA approval.

In this paper, we survey the research published from January 2020 through March 2021 to identify the current challenges to meaningful use of AI in transplant, and opportunities to advance and prepare for AI deployment.

### AI for Transplant

Articles discussing AI often do not define the term, and there are a wide variety of definitions offered by those that do. Drawing on work in the field of human-computer interaction, a useful definition of artificial intelligence for transplant is *an information system capable of considering data and making clinical or patient care decisions commonly associated with a human*.^1^ Having an appropriately broad definition allows for a wide variety of AI applications and new developments in the field.

The term AI often prompts people to think of the fictional technologies depicted in movies or literature such as humanoid robots or disembodied assistants; these agents are examples of general purpose artificial intelligence. In reality, most AI agents will address a handful of tasks (eg, Tesla’s Autopilot system drives the car but cannot hold a conversation). Rather than an “AI Transplant Nephrologist,” we anticipate individual AI tools that address a single or small subset of clinical considerations that drive care, such as predicting waitlist time or rejection risk, or making recommendations for immunosuppression dosing.

AI decisions are enacted without human intervention in many fields. For example, Uber’s algorithms match drivers and riders without a human manager approving each transaction. However, legal, moral, and ethical dilemmas arise in even relatively “benign” AI applications, so we do not anticipate many (if any) transplant decisions being made by AI alone for the foreseeable future. For many years to come, coordinating patient care and making final decisions is the exclusive domain of human professionals. AI tools will operate with a “human in the loop,” augmenting human decisionmakers.

There are several excellent reviews of how AI may be applied in transplant (7, 8), and about how different AI methods work (9). Briefly, two broad types of AI relevant to transplant are rules-based expert systems and machine learning systems. Rules-based systems are a kind of AI that relies on the knowledge and practice of experts and essentially consist of a decision tree with many if/then statements. Healthcare applications of expert systems have existed since the 1970s (10), and many modern clinical decision support systems can be considered (simple) expert systems.

In contrast, machine learning systems are AI agents which “learn” from a training dataset instead of being explicitly programmed. Supervised learning is a subset of ML in which labeled data (eg, patients with rejection vs. no rejection) is given to the program so that it can learn to predict rejection; supervised learning methods include logistic regression^2^, decision trees, random forest, and neural networks among others. Unsupervised learning is another subset of ML; it uses algorithms to discover patterns or clusters within the data (eg, patient “types” derived from an attitudes survey).

Current technology, including free open-source software, is capable of making some excellent clinical predictions; many of the studies we identified in our review show an ML or AI tool outperforming the current state-of-the-art (e.g. the MELD score).

## Method

To provide a timely update about the state of challenges to implementing AI in transplant, we conducted a restricted systematic review (RSR, sometimes called a rapid review) to evaluate literature published in 2020-2021. RSRs allow for the timely synthesis of information but make concessions with regard to the amount of time and number of sources sought, and the number of reviewers evaluating each publication. However, RSRs are widely used and have been shown to generate similar findings to systematic reviews (11).

**Figure 1:**
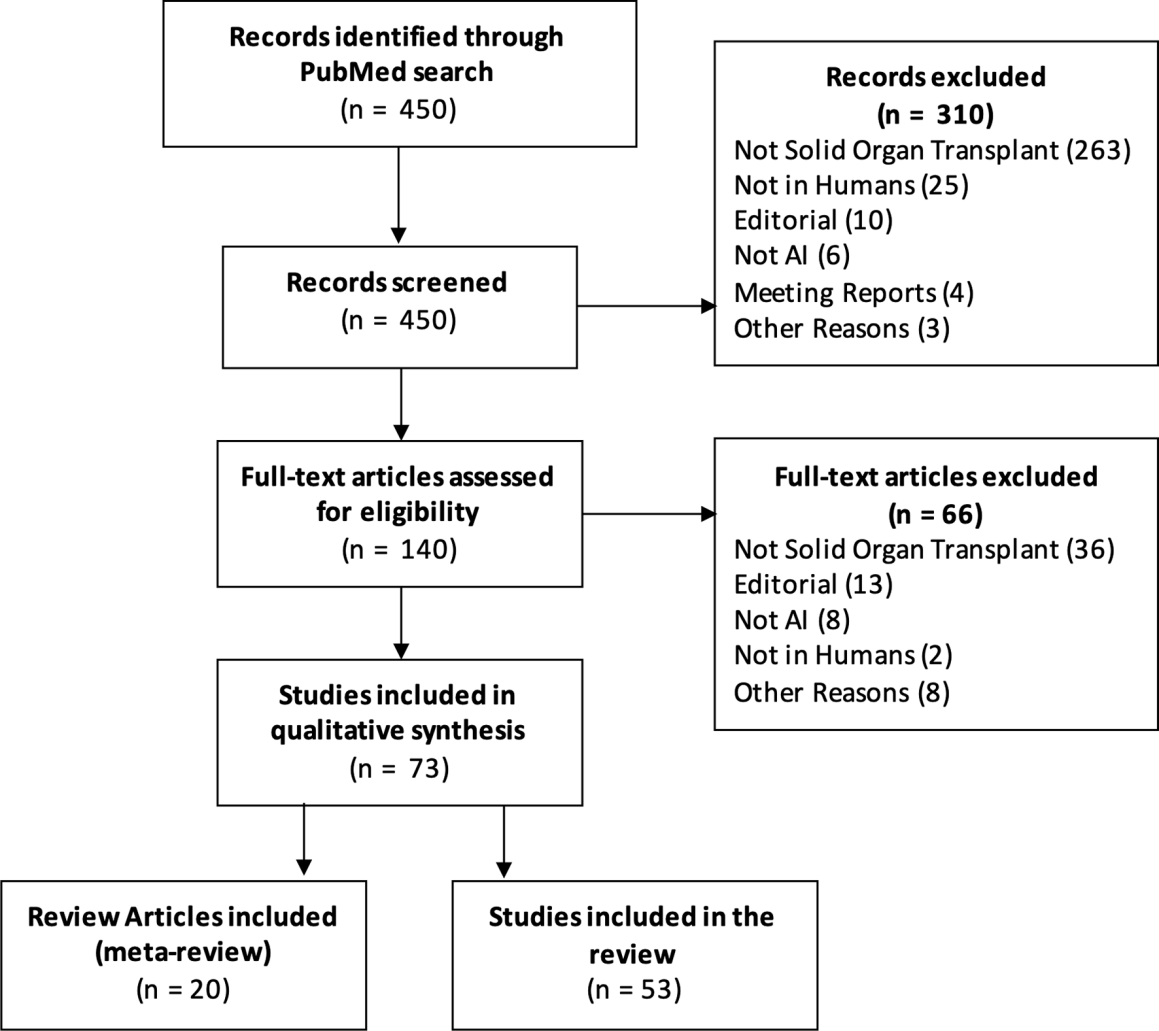
PRISMA Diagram detailing the selection and screening of records included in the review.

We searched the PubMed database with the query “(transplant artificial intelligence) OR (transplant machine learning) OR (transplant neural net) OR (transplant random forest) OR (transplant nlp) OR (transplant svm)” and filtered results published in 2020 or 2021 (through 10 Mar 2021). **Figure 1** is a PRISMA diagram detailing the screening and selection process. Out of 450 initial results, 74 articles met the inclusion criteria.

Each article was read and coded to capture the limitations/challenges highlighted by the authors, and, if a study, the AI prediction/recommendation being made, the AI methods used, and the effectiveness/accuracy methods reported.

## Findings and Emergent Themes

Our search identified 20 review articles covering transplant-related artificial intelligence. These articles varied in their scope (some focused on AI methods, others focused on specific applications such as pathology or donor matching) and their method (e.g. systematic, scoping, and state-of-the-art review methods). We also identified 54 studies which developed an AI model or models. **Table 1** summarizes the challenges and limitations identified, the AI methods used, and the effectiveness criteria reported. Full details for each study are provided in **Supplementary Table 1**.

**Table 1:** AI Methods, Effectiveness and Accuracy Criteria, and Challenges identified by studies in review.

Category	Method/Criteria (n = Records Reporting)
AI Methods Used (Studies Only)	Random Forest (n = 24)
	Neural Networks (n = 18)
	Gradient Boosting (n = 11)
	Logistic Regression (n = 9)
	Decision Trees (n = 7)
	Support Vector Machine (n = 7)
	kNN (n = 3)
	LASSO or Ridge Regression (n = 3)
	Natural Language Processing (n = 3)
	Adaptive Boosting (Adaboost) (n = 2)
	Naïve Bayes (n = 2)
	Other or Unspecified Method (n = 8)
AI Effectiveness and Accuracy Criteria Reported (Studies Only)	Area Under ROC (AUC) (n = 21)
	Sensitivity (n = 17)
	Specificity (n = 12)
	Accuracy (n = 13)
	Precision (n = 4)
	Recall (n = 2)
	C-Index (n = 12)
	F1 (n = 3)
	Brier Score (n = 3)
	Positive Predictive Value (n = 6)
	Negative Predictive Value (n = 5)
	Cost/Benefit Metric (n = 2)
	RMSE (n = 2)
	Custom Metric (n = 1)
	None Reported (n = 9)
	Other (n = 11)
Challenges and Limitations Highlighted (Studies and Reviews)	Data
	Generalizable/Representative Data (n = 48)
	Collection/Measurement (n = 7)
	Missing data (n = 6)
	Clinical Decision Process
	Interpretability/Explanation (n = 13)
	Clinician Training on Use of AI (n = 4)
	Acceptability
	Validation/Approval (n = 16)
	Ethical Guidelines (n = 8)
	Accuracy/Acceptance Criteria (n = 4)
	Commercial Vested Interested (n = 2)

### AI Methods Utilized

As there is no “best” AI or machine learning method, many studies utilized multiple AI methods and compared their performance. Random forest methods were the most popular, followed by neural networks. It is important to note that there are many variations and subsets within a given model, and most models have a variety of user-selectable inputs as well as parameters that can be optimized based on user-selected criteria. In other words, two different researchers might develop very different models even though they utilize the same data set and the same basic ML method. The key takeaway is that researchers are exploring a variety of methods, but the most popular methods are not easily interpretable or explainable—they are a black box.

### Accuracy and Effectiveness Measures Reported

Studies reported a wide range of metrics to evaluate the effectiveness or accuracy of the tools they developed, with area under the Receiver Operator Curve (AUC or AUROC) being the most common. This lack of standardization makes it difficult to compare results between studies. We further note that not all studies compare the AI tools they have developed to the status quo “non-AI tool” (e.g. MELD or SOFT scores) (12). Even so, evaluating accuracy, sensitivity, specificity, the Receiver Operator Curve (ROC) AUC, etc., will only provide insight to how well an AI makes decisions against retrospective data. Two studies reported a cost/benefit metric of some kind, indicating the potential value and quantifying the tradeoffs of using the tool.

At a minimum, we recommend authors include AUC, accuracy, sensitivity, specificity, and compare model performance to the status quo (if applicable) but acknowledge that different situations and models warrant different metrics. However, only reporting a single metric (e.g. accuracy) should be avoided.

### Current Challenges to Implementing AI in Transplant

From the literature we reviewed, we identified three main challenges to adopting AI agents in transplant: having suitable data, incorporating algorithms into decisions, and acceptability criteria for adoption.

#### Challenge #1: Data Availability and Bias

The most common challenge, identified by 48 articles, was a lack of the generalizable, representative data to train the algorithm such that it would work well on the general patient population; the problem is exacerbated by data sets with large proportions of records with missing data. The training data can also lead to biased predictions for certain patients if they are un- or under-represented in the training dataset (13). In transplant, it is possible that difficult cases, comorbidities and patients receiving novel treatments will be un- or under-represented in any training data, but these are the patients who may benefit most from a personalized treatment recommendation (14). Generating predictions also requires data on the current patient case to be evaluated by the model; seven articles noted that the data collected on cases to be predicted may vary somehow from the training data (e.g. due to local practices) which would result in inaccurate predictions.

A comprehensive transplant database does not currently exist. The current available “big data” repositories in transplantation include Medicare and pharmacy claims databases, the Scientific Registry of Transplant Recipients, and large-scale clinical trial databases, which are expansive in and of themselves but do not contain a complete dataset of which all desired data points are captured or well-described. In particular, these datasets may not be representative of the patient population in a given community or hospital which can potentially lead to some of the issues discussed above.

Training data may never be perfect, and the data that the system uses to make predictions about a new patient will not undergo rigorous validation while the patient and clinician wait. Successful use of AI tools will require a validation process to certify that a model is “good enough” based on the data available and provides more benefit than harm. And just as with other diagnostic and decision tools, as clinicians gain experience with a model they will become attuned to when the model might be wrong.

#### Challenge #2: The Clinical Decision Process and AI Explainability

A second frequently identified challenge to adopting AI relates to uncertainty with how to incorporate AI recommendations into clinical decisions. In particular, the “black box” nature of many AI algorithms is noted in 13 articles in our review. While some simpler methods like decision trees or logistic regression are “explainable” in that you can see which patient features are driving a decision, it is not usually possible to provide an interpretable explanation for the recommendation from most common, complex AI methods like neural networks or random forest. For example, the hidden layers of a neural network—key to its operation—do not necessarily correspond to any physiological process.

The lack of interpretability is particularly problematic since AI can only have an impact when it helps people arrive at a better decision than they would without the AI—they must be adjusting their decision. If the AI recommendation always aligns with what people would do anyway (or are already doing), outcomes are no different than they would be otherwise. Thus, incorporating AI recommendations (often without an interpretable explanation for where the recommendation is coming from) into clinical decisions is a new skill for which there are no guidelines or textbooks (6, 15).

There is some research on developing methods similar to sensitivity analysis that can provide explanations from black box AI tools, but these methods introduce another layer with its own uncertainty. Additionally, there is some evidence that providing explanations for AI recommendations is not always perceived as useful or impactful, and can potentially lead clinicians to ignore their own best judgment when the AI is incorrect (16).

#### Challenge #3: AI Acceptability – Clinical and Ethical Considerations

When is an AI tool good enough to use? When is it ethical to consult an AI? 16 papers noted the need for prospective evaluation or a regulatory approval process. Authors also identified the need for ethical guidelines for incorporating AI recommendations and standardized accuracy criteria. For a commercial firm to sell a clinical AI agent in the United States, they will generally have to receive FDA approval for the tool under the Software as a Medical Device (SaMD) protocol, but the same is not necessarily true of “home grown” AI tools. For example, a hospital would not be required to follow the process before implementing an in-house tool used to stratify patients based on their risk for medication non-adherence if it were for research purposes. On the one hand, there will be tools that undergo rigorous testing and prospective evaluation over a period of years, across multiple published studies, in coordination with the FDA and with transparency among the transplant community; on the other hand, one can imagine that there will be smaller niche or commercial tools that do not undergo such testing (17).

## Discussion

### Toward Meaningful Use of AI in Transplant

Drawing on the definition of meaningful use of electronic health records, we define meaningful use of AI as the application of AI to guide clinical decision making and improve patient outcomes in transplantation. Most of the algorithms that have been developed in transplant only ever touch retrospective data and are never put into clinical practice. Reaching meaningful use of AI in transplant is an interdisciplinary challenge that requires careful work and requires practice, discussion, and research on issues that can begin immediately.

#### At the Transplant Center Level: Assemble a Transplant AI Team

Just as Quality Improvement is most effective when conducted by a team with a deliberate process, AI deployment should be done by a team potentially consisting of clinicians, administrators, AI experts, and ethicists. Every center should consider assembling a Transplant AI team spearheaded by an “AI Champion” to evaluate AI tools as they become available (or to coordinate the development of in-house tools as needs are identified). QI is a learned skill, and we anticipate that AI evaluation, deployment and adoption is likely to be a learned skill as well. **Supplementary Table 2** lists some individuals to consider including on the Transplant AI team; the exact composition of the team may fluctuate based on the project, but members might include surgeons, nephrologists and hepatologists, infectious disease specialists, pharmacists, nurses, hospital administrators, data analytics personnel, biostatisticians, human-computer interaction researchers, and ethicists.

Although many pre-built AI systems exist (or can be purchased), it will often be beneficial to bring in an expert to assist with developing or evaluating an AI tool. A university’s biostatistics division is also a good place to start a discussion, but experts willing to collaborate are often available across any research university (e.g., within the statistics, computer science, data science, or information systems departments).

#### At the Field Level: Establishing Clinical and Ethical Acceptability Criteria

When is an AI good enough? Currently, there is no agreement about how to evaluate an AI for adoption. It may be worthwhile for journals to set a minimum standard for evaluation metrics reported for AI methods, and a valuable contribution with any AI paper will be to compare the results from the new model to the current standard of practice.

Whether an AI is acceptable to put into practice is a function of how accurate it is for a given patient population, and the potential benefit it offers over the status quo (of an unaided human decisionmaker) weighed against the downside risk if the AI is incorrect. The field of transplant would benefit from having criteria or guidelines to assist with these decisions. One metric to consider is the degree to which human experts agree with the AI’s predictions; this metric has been widely used to evaluate AI, including in transplant (18). However, any cutoff for human agreement is arbitrary and just because experts agree with the AI does not mean it is right; likewise, disagreement with the AI does not mean that it is incorrect in a given case. Another metric to consider is the accuracy of the AI compared to the typical human decisionmaker. Finally, the cost/benefit of the AI should be considered, the calculation of which will vary based on context.

#### At the Field Level: Incorporate AI Into the Shared Decision Model

The Shared Decision Making (SDM) Model involves the patient in clinical decisions (19). The SDM offers potential benefits in transplant care because of the chronic nature of care, the complexity of the condition, and the role of the patient in managing their own care, but how AI is incorporated into shared decisions is unknown. When and how should you discuss the clinical team’s use of AI with patients? Consider a patient who is asking questions about their current immunosuppression regimen (eg, the need for steroids or current tacrolimus dose). It is unknown how a given patient will respond if they are told that AI was a factor in a clinical decision (such as which immunosuppressant is recommended). Perhaps sharing that an AI trained on millions of datapoints concurs with a recommendation will help improve buy-in (20). On the other hand, there is evidence that people trust doctors less when they rely on expert-based AI systems, so revealing that AI was consulted may reduce patient trust and buy-in (21).

There are also questions about ethical requirements to disclose when AI was utilized. However, we note that ethical norms do not require clinicians to disclose when they sought a second opinion from a colleague.

## Conclusion

The transplant AI research that was published in the past year continues to demonstrate the potential benefits that AI can offer to improve patient outcomes, but the challenges and limitations identified are ones that will not be resolved with better AI technology. To move AI forward, we encourage centers to begin engaging with it, and transplant researchers to include implementation considerations in future AI studies. Finally, debating and coming to some consensus on AI acceptability guidelines is an essential conversation the field must have over the coming years. The conversation will move forward rapidly if assisted by venues such as special issues of journals, workshops and roundtables in Communities of Practice, and plenary sessions at conferences.

## Author Contributions

JC conducted the literature review. All authors contributed to the article and approved the submitted version. All authors contributed to the article and approved the submitted version.

## Conflict of Interest

AM is employed as a Scientific Affairs Director at Hansa Biopharma AB.

The remaining author declares that the research was conducted in the absence of any commercial or financial relationships that could be construed as a potential conflict of interest.

## Footnotes

1 For example, see Merriam-Webster’s definition of AI (https://www.merriam-webster.com/dictionary/artificial%20intelligence).

2 Logistic regression is included in many standard machine learning software packages, but whether it is a “statistical technique” or “machine learning method” is the subject of some debate. Logistic regression models often perform well, require comparatively less computing resources, and are interpretable.

## References

[1] Zarrinpar A Lee DK Silva A Datta N Kee T Eriksen C. Individualizing Liver Transplant Immunosuppression Using a Phenotypic Personalized Medicine Platform. Sci Transl Med (2016) 8(333):333ra49. 10.1126/scitranslmed.aac5954 27053773

[2] Fu S Zarrinpar A. Recent Advances in Precision Medicine for Individualized Immunosuppression. Curr Opin Organ Transplant (2020) 25(4):420–5. 10.1097/MOT.0000000000000771 PMC7723319 32520785

[3] Dawes RM Faust D Meehl PE. Clinical Versus Actuarial Judgment. Science (1989) 243:1668–74. 10.1126/science.2648573 2648573

[4] Sekercioglu N Fu R Kim SJ Mitsakakis N. Machine Learning for Predicting Long-Term Kidney Allograft Survival: A Scoping Review. Ir J Med Sci (2021) 190(2):807–17. 10.1007/s11845-020-02332-1 32761550

[5] Bae S Massie AB Caffo BS Jackson KR Segev DL. Machine Learning to Predict Transplant Outcomes: Helpful or Hype? A Natl cohort study Transpl Int (2020) 33(11):1472–80. 10.1111/tri.13695 PMC8269970 32996170

[6] Cresswell K Callaghan M Khan S Sheikh Z Mozaffar H Sheikh A. Investigating the Use of Data-Driven Artificial Intelligence in Computerised Decision Support Systems for Health and Social Care: A Systematic Review. Health Inf J (2020) 26(3):2138–47. 10.1177/1460458219900452 31964204

[7] Spann A Yasodhara A Kang J Watt K Wang B Goldenberg A. Applying Machine Learning in Liver Disease and Transplantation: A Comprehensive Review. Hepatology (2020) 71(3):1093–105. 10.1002/hep.31103 31907954

[8] Díez-Sanmartín C Sarasa Cabezuelo A. Application of Artificial Intelligence Techniques to Predict Survival in Kidney Transplantation: A Review. J Clin Med (2020) 9(2):572. 10.3390/jcm9020572 PMC7074285 32093027

[9] Coorey CP Sharma A Muller S Yang JYH. Prediction Modeling—Part 2: Using Machine Learning Strategies to Improve Transplantation Outcomes. Vol 99 Kidney Int Elsevier B.V.; (2021) p:817–23. 10.1016/j.kint.2020.08.026 32916179

[10] Buchanan BG Shortliffe EH. Rule-Based Expert Systems: The Mycin Experiments of the Stanford Heuristic Programming Project (Addison-Wesley Series in Artificial Intelligence). Reading, Mass: Addison-Wesley (1984).

[11] Plüddemann A Aronson JK Onakpoya I. Redefining Rapid Reviews: A Flexible Framework for Restricted Systematic Reviews An Operational Definition of a Restricted Review in a Flexible Framework EBM Opinion and Debate. BMJ Evidence-Based Med (2018) 23(6):201–3. 10.1136/bmjebm-2018-110990 29950313

[12] Wingfield LR Ceresa C Thorogood S Fleuriot J Knight S. Using Artificial Intelligence for Predicting Survival of Individual Grafts in Liver Transplantation: A Systematic Review. Liver Transplant (2020) 26(7):922–34. 10.1002/lt.25772 32274856

[13] Gianfrancesco MA Tamang S Yazdany J Schmajuk G. Potential Biases in Machine Learning Algorithms Using Electronic Health Record Data. JAMA Intern Med (2018) 178(11):1544–7. 10.1001/jamainternmed.2018.3763 PMC6347576 30128552

[14] Chen IY Szolovits P Ghassemi M. Can AI Help Reduce Disparities in General Medical and Mental Health Care? AMA J Ethics (2019) 21(2):167–79. 10.1001/amajethics.2019.167 30794127

[15] Becker JU Mayerich D Padmanabhan M Barratt J Ernst A Boor P. Artificial Intelligence and Machine Learning in Nephropathology. Kidney Int [Internet] (2020) 98(1):65–75. 10.1016/j.kint.2020.02.027 PMC8906056 32475607

[16] Bussone A Stumpf S O’Sullivan D. The Role of Explanations on Trust and Reliance in Clinical Decision Support Systems. Proc - 2015 IEEE Int Conf Healthc Inf ICHI (2015) 2015:160–9. 10.1109/ICHI.2015.26

[17] Benjamens S Dhunnoo P Meskó B. The State of Artificial Intelligence-Based FDA-approved Medical Devices and Algorithms: An Online Database. NPJ Digit Med [Internet] (2020) 3(1):1–8. 10.1038/s41746-020-00324-0 PMC7486909 32984550

[18] Reeve J Böhmig GA Eskandary F Einecke G Gupta G Madill-Thomsen K. Generating Automated Kidney Transplant Biopsy Reports Combining Molecular Measurements With Ensembles of Machine Learning Classifiers. Am J Transplant [Internet] (2019) 19(10):2719–31. 10.1111/ajt.15351 30868758

[19] Légaré F Stacey D Turcotte S Cossi MJ Kryworuchko J Graham ID. Interventions for Improving the Adoption of Shared Decision Making by Healthcare Professionals ( Review ) SUMMARY of FINDINGS for THE Main Comparison. Cochrane Database Syst Rev (2014) 15(9):CD006732. 10.1002/14651858.CD006732.pub3 25222632

[20] Osterberg L Blaschke T. Adherence to Medication. N Engl J Med (2005) 353:487–97. 10.1056/NEJMra050100 16079372

[21] Arkes HR Shaffer VA Medow MA. Patients Derogate Physicians Who Use a Computer-Assisted Diagnostic Aid. Med Decis Mak (2007) 27:189–202. 10.1177/0272989X06297391 17409368

