# Supplementary material for: Augmenting the Transplant Team With Artificial Intelligence: Toward Meaningful AI Use in Solid Organ Transplant
Source: Front Immunol. 2021 Jun 11;12:694222. doi: 10.3389/fimmu.2021.694222 (PMC8226178; doi:10.3389/fimmu.2021.694222)
Supplement: Supplementary file 2 [file DataSheet_1.pdf]

Team Member	Role
Transplant Surgeons	Clinical and Practice Expertise
Transplant Nephrologists, Hepatologists, Infectious Disease Specialists, etc.	Clinical and Practice Expertise
Transplant Pharmacist	Clinical and Practice Expertise
Transplant Nurses	Clinical and Practice Expertise
EHR Administrators/Support	EHR Integration, Model deployment
Hospital Data Analytics Team	Enterprise Data, HIPAA Considerations
Hospital Administration	Policy, Legal, Financial Considerations
Data Scientists, Biostatisticians, AI Developers	AI Model Development
Information Systems (IS) or Human-Computer Interaction (HCI) Researchers	Expertise developing recommender systems and studying how AI recommendations are incorporated into decision processes
Ethicist	Ethics and patient representation

Supplementary Table 2: Possible Transplant AI Team Composition: The team should be adjusted depending on the transplant specialty and hospital organization. Additional members should be added or invited as appropriate.
